# Recurrent Cervical Esophageal Fistula and Retroesophageal Abscess Following Surgical Management of Zenker’s Diverticulum

**DOI:** 10.3390/jcm15072777

**Published:** 2026-04-07

**Authors:** Bogdan Mihnea Ciuntu, Andreea Ludusanu, Mara Teodora Zara, Mihaela Corlade-Andrei, Adelina Tanevski, Cristinel Ionel Stan, Dragos Andrei Chiran, Dan Vintila, Dan Andronic, Gheorghe Balan

**Affiliations:** Faculty of Medicine, Grigore T. Popa University of Medicine and Pharmacy Iasi, University Street No. 16, 700115 Iasi, Romania; bogdan-mihnea.ciuntu@umfiasi.ro (B.M.C.); mihaela.corlade2@umfiasi.ro (M.C.-A.); papancea.adelina@umfiasi.ro (A.T.); cristinel.stan@umfiasi.ro (C.I.S.); dragos-andrei.chiran@umfiasi.ro (D.A.C.); dan.vintila@umfiasi.ro (D.V.); dan.andronic@umfiasi.ro (D.A.); gheorghe-g-balan@umfiasi.ro (G.B.)

**Keywords:** Zenker’s diverticulum, cervical esophageal fistula, extraluminal VAC, vacuum-assisted closure, minimally invasive therapy

## Abstract

**Background**: Zenker’s diverticulum arises from the posterior hypopharyngeal wall through Killian’s dehiscence and predominantly affects older adults. Surgical and endoscopic treatments may be complicated by adverse events, including recurrent laryngeal nerve injury, cervical emphysema, mediastinitis, and pharyngoesophageal fistula formation. **Methods**: We report the case of a 69-year-old male who underwent open surgical treatment for Zenker’s diverticulum and subsequently developed an upper esophageal fistula complicated by a retroesophageal abscess. **Results**: The patient was treated using an externally adapted endoluminal vacuum-assisted closure system (EndoVAC), which enabled continuous drainage, local lavage, and progressive closure of the esophageal defect. **Conclusions**: Endo-VAC therapy represents a safe and minimally invasive therapeutic option for the management of postoperative esophageal fistulas following Zenker’s diverticulum surgery and may reduce the need for extensive esophageal reconstruction.

## 1. Introduction

Zenker’s diverticulum (ZD) is a pulsion diverticulum arising from the posterior wall of the hypopharynx through Killian’s triangle, an anatomically weak area located between the oblique fibers of the thyropharyngeal muscle and the horizontal fibers of the cricopharyngeal muscle [1]. Although considered a rare entity, ZD represents the most common type of esophageal diverticulum, accounting for approximately 75% of all esophageal diverticula [2]. It predominantly affects elderly patients, most frequently during the seventh and eighth decades of life, and can lead to significant morbidity manifested by dysphagia, regurgitation, pulmonary aspiration, chronic cough, and weight loss [3].

Anatomical studies have demonstrated a left-sided predominance of ZD, which is thought to result from asymmetry of the pharyngeal musculature, with a thinner protective muscular layer on the left side predisposing to preferential herniation [4,5]. Symptomatic ZD, particularly large diverticula or those associated with significant clinical impairment, usually requires intervention. Traditional management consists of open transcervical surgery, including diverticulectomy or diverticulopexy combined with cricopharyngeal myotomy, which remains a well-established and effective approach [6]. Postoperative hypopharyngeal and cervical esophageal fistulas remain rare but potentially severe complications after Zenker’s diverticulectomy. Recent literature emphasizes minimally invasive and endoscopic strategies aimed at reducing morbidity, including stenting, clipping, and endoluminal vacuum therapy. However, management becomes challenging when extraluminal abscess cavities coexist [4,7].

Despite technical advancements, both open surgical and endoscopic treatments for ZD carry inherent risks. Open surgery may be associated with complications such as cervical hematoma, recurrent laryngeal nerve injury, mediastinitis, and esophageal perforation [8]. Endoscopic therapies generally have a more favorable safety profile; however, adverse events may still occur, including perforation, subcutaneous emphysema, bleeding, infection, and mediastinitis [9]. Among the most challenging complications is the development of postoperative leaks or fistulas, which have traditionally required surgical reintervention.

Advances in therapeutic endoscopy have expanded the available treatment options for esophageal leaks, perforations, and fistulas of the upper gastrointestinal tract. Commonly employed endoscopic modalities include self-expandable metal stents, endoscopic clips, suturing systems, and other closure devices, although their use may be limited in certain clinical scenarios [10]. In this context, endoluminal vacuum-assisted closure (EndoVAC) therapy has emerged as a novel and promising salvage strategy for the management of esophageal leaks and fistulas [11]. By providing continuous drainage, reducing local contamination, and promoting granulation tissue formation, VAC therapy facilitates progressive defect closure while avoiding the morbidity associated with repeat open surgical procedures [12,13].

In this article, we present a case of a recurrent cervical esophageal fistula associated with a retroesophageal abscess following surgical resection of a Zenker’s diverticulum. In this patient, a modified vacuum-assisted closure system was externally adapted to the fistulous site to enable effective local drainage and promote healing of the esophageal defect. Conventional endoluminal approaches—including stenting, clipping, and endoluminal VAC therapy—were not feasible; therefore, the externally applied VAC system proved to be a valuable alternative by establishing controlled external drainage and facilitating progressive closure of the esophageal wall.

## 2. Case Presentation

A 69-year-old male presented to the Emergency Department of “Sf. Spiridon” County Emergency Hospital in Iași with progressive dysphagia and foul-smelling regurgitation of six months’ duration. A prior diagnostic work-up had established the presence of Zenker’s diverticulum based on contrast-enhanced computed tomography and upper gastrointestinal endoscopy. On admission, the patient was hemodynamically stable. Laboratory tests showed mildly elevated inflammatory markers, without other significant abnormalities (Table 1).

**Figure 1:**
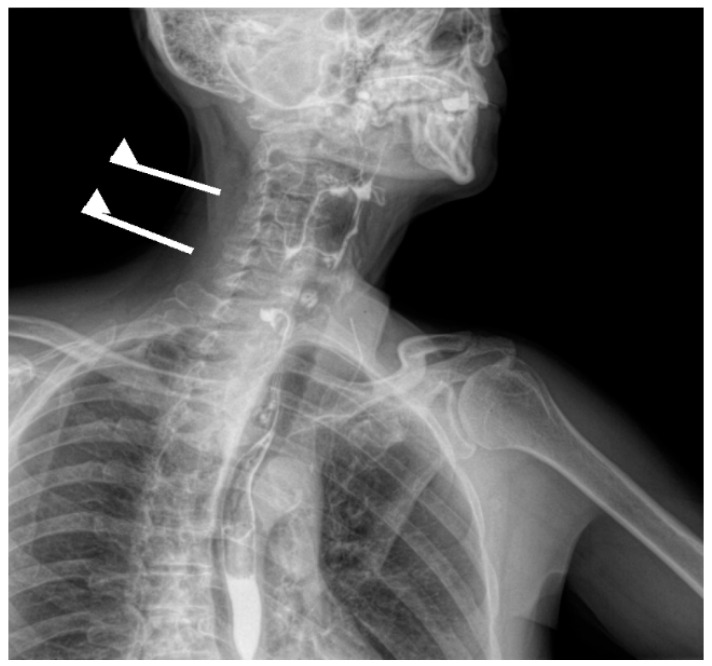
Lateral contrast esophagography demonstrating a posterior contrast-filled outpouching arising from the pharyngoesophageal junction at approximately the C5–C6 level, consistent with Zenker’s diverticulum.

**Figure 2:**
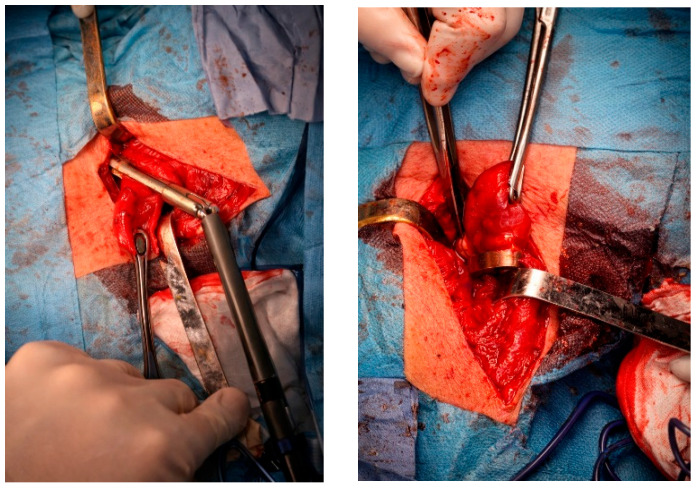
Intraoperative view demonstrating diverticulectomy with mechanical closure of the esophageal defect using a linear stapler. The diverticulum neck is positioned between the stapler cartridge and anvil, allowing simultaneous resection and creation of a mechanical staple line along the esophageal wall. Retractors maintain exposure of the cervical operative field, while suction and electrocautery instruments assist in visualization, tissue dissection, and hemostasis.

Contrast-enhanced esophagogastroduodenal radiography confirmed a 57 × 66 mm cervicothoracic diverticulum located posterior to the esophageal wall (Figure 1). After multidisciplinary evaluation and preoperative assessment, open diverticulectomy with linear stapler closure of the esophageal defect and cervical drainage was performed (Figure 2). Following open diverticulectomy (postoperative day [POD] 0), the initial postoperative course was uneventful. A contrast study on postoperative day five showed no evidence of leakage. The patient was discharged in good clinical condition, tolerating oral intake.

**Figure 3:**
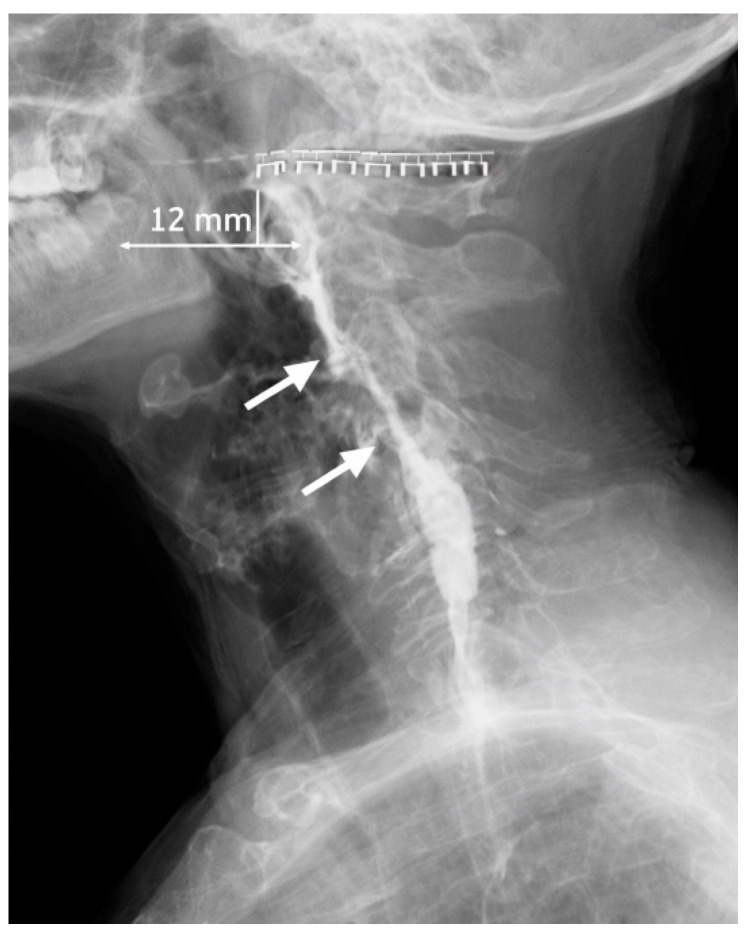
Lateral contrast esophagography demonstrating a contrast-filled fistulous tract (arrows) originating approximately 12 mm inferior to the superior margin of the mechanical staple line following diverticulectomy.

On POD 15, he was readmitted with dysphagia, left cervical pain, fever, and laboratory evidence of systemic inflammation. Contrast examination with Barium sulfate demonstrated a 12 mm fistulous tract originating from the superior aspect of the staple line, consistent with a postoperative cervical esophageal fistula (Figure 3). Surgical wound exploration with drainage and lavage was undertaken, and empirical broad-spectrum antibiotic therapy was initiated.

**Figure 4:**
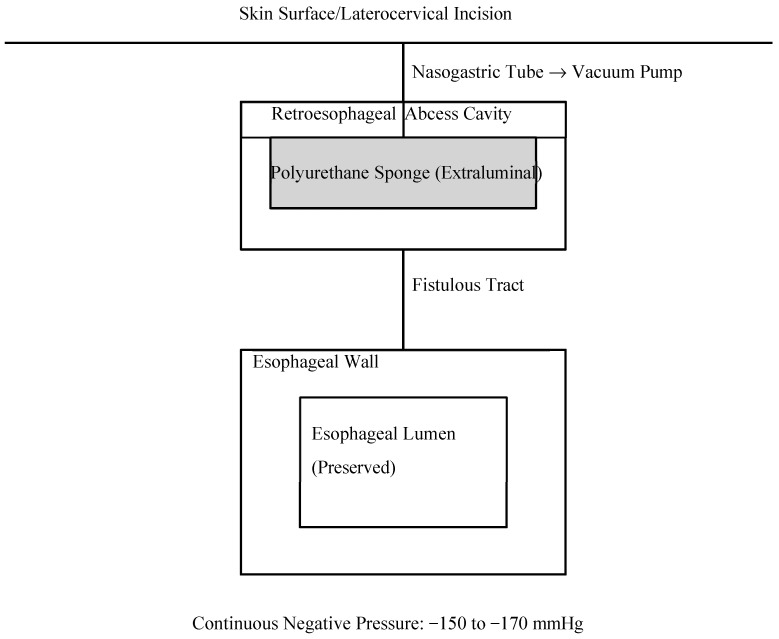
Schematic representation of externally adapted vacuum-assisted closure (VAC) therapy for cervical esophageal fistula. The polyurethane sponge is positioned extraluminally within the fistulous tract via a laterocervical incision. A nasogastric tube embedded within the sponge is connected to continuous negative pressure (−150 to −170 mmHg). The esophageal lumen remains preserved, allowing controlled oral intake during therapy.

**Figure 5:**
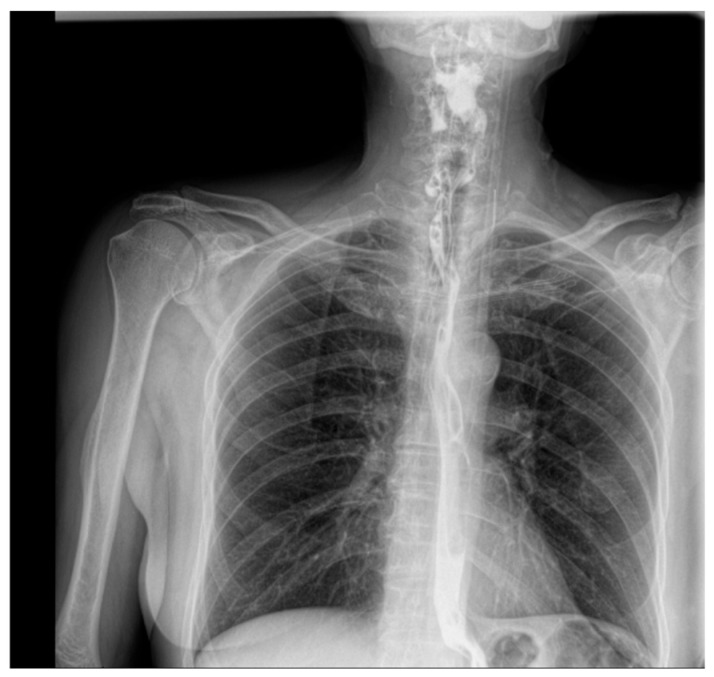
Follow-up contrast esophagography demonstrating a continuous contrast column within the esophagus after surgical treatment.

Given the presence of a retroesophageal collection and anatomical limitations precluding conventional endoluminal techniques, a customized externally adapted vacuum-assisted closure (VAC) system was placed via left lateral cervicotomy under endoscopic guidance (Figure 4). Continuous negative pressure (150–170 mmHg) was applied for 11 days, with one sponge exchange at 6 days, criteria for discontinuation: absence of purulent drainage, progressive reduction in cavity size, granulation tissue formation, and radiological confirmation of fistula closure. Microbiological cultures identified Streptococcus anginosus, allowing targeted antibiotic therapy. Radiological closure was confirmed after 11 days of VAC therapy. Clinical, laboratory, and radiological parameters progressively improved, and the patient was discharged with satisfactory wound healing and tolerance of a liquid diet (Figure 5).

**Figure 6:**
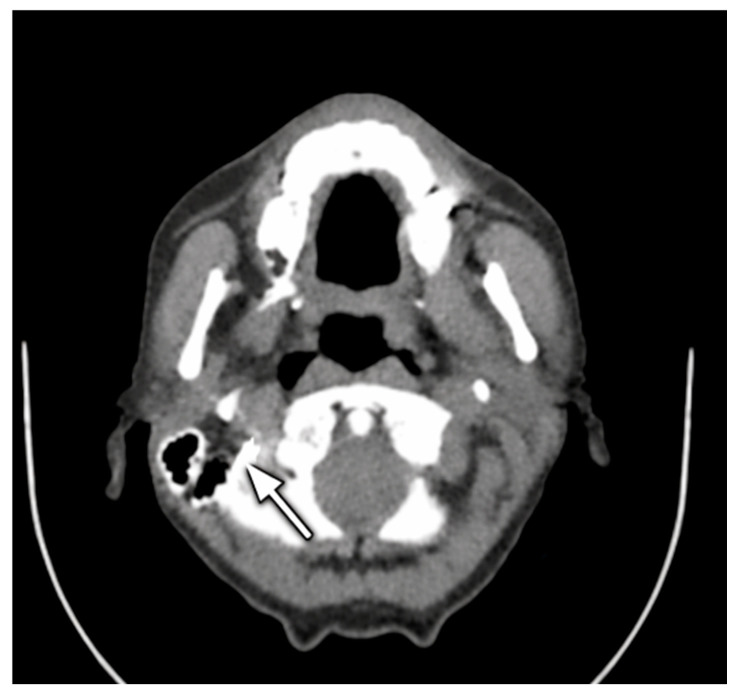
Axial contrast-enhanced CT scan of the neck showing a retroesophageal abscess with intralesional air bubbles. The arrow indicates the suspected cervical esophageal fistulous tract communicating with the collection.

During subsequent follow-up, two additional readmissions occurred due to recurrent cervical discomfort and inflammatory episodes. Upon the third readmission Ct imaging (Figure 6) demonstrated a retroesophageal abscess with minimal contrast extravasation. Contrast-enhanced CT plays a central role in the diagnosis of cervical esophageal fistulas and retroesophageal abscesses, allowing precise localization of extraluminal collections, evaluation of mediastinal extension, and guidance for drainage or surgical planning. At the time of the fourth readmission, the patient appears to be in a state of significant systemic inflammation consistent with ongoing infection, likely related to the retroesophageal abscess and persistent cervical septic focus (Table 2). Targeted cervical incision, evacuation, lavage, and drainage were performed, resulting in favorable clinical evolution.

At final follow-up, no evidence of persistent fistula or abscess was observed. Conservative management of minor superficial inflammatory changes led to complete resolution.

## 3. Discussion

This report does not aim to introduce a new therapeutic principle but rather to describe a context-specific adaptation of established negative-pressure therapy concepts in a cervical extraluminal setting where conventional endoluminal strategies were insufficient. The contribution of this case lies in defining anatomical and clinical conditions under which such an adaptation may be considered.

Traditional surgical approaches for esophageal fistula repair, including muscle flap reconstruction or esophageal exclusion, are associated with considerable morbidity, particularly in elderly patients and in the post-esophagectomy setting [13,14]. In recent years, endoluminal vacuum-assisted closure (EndoVAC) therapy has gained wide acceptance as a minimally invasive alternative, demonstrating high clinical success rates in the manage-ment of esophageal leaks and fistulas [14,15].

Although endoluminal vacuum therapy (EndoVAC) is well established for the management of esophageal leaks and anastomotic dehiscence, reports specifically describing an externally adapted extraluminal vacuum configuration for cervical esophageal fistulas following Zenker’s diverticulum surgery are scarce [15]. In the available literature, vacuum therapy is predominantly applied intraluminally, targeting thoracic anastomotic leaks or postoperative perforations [15,16]. The present case therefore does not introduce a fundamentally new therapeutic principle but rather describes a technical adaptation of established negative-pressure therapy concepts to a cervical extraluminal setting, where classical endoluminal approaches were not feasible.

From a pathophysiological perspective, persistent cervical esophageal fistulas may result from impaired local perfusion, excessive tissue tension, and maintenance of a septic extraluminal cavity [16]. In this case, recurrence likely reflected incomplete collapse of the retroesophageal abscess cavity rather than the formation of a de novo defect. Classical intraluminal approaches primarily address mucosal sealing but may fail to adequately drain associated extraluminal collections. The application of negative pressure directly within the cervical abscess cavity may have promoted cavity collapse, improved local microcirculation, reduced bacterial load, and stimulated granulation tissue formation, thereby facilitating progressive closure.

The present case highlights a modified therapeutic strategy using an externally adapted vacuum-assisted closure system, which represents a distinct and innovative variation in conventional EndoVAC therapy. Unlike the classical endoluminal approach, the vacuum sponge in our technique was positioned extraluminally within the fistulous tract via a lateral cervical access. This configuration preserved the esophageal lumen and avoided direct contact with the intraluminal suture line, thereby reducing the risk of further mucosal injury, anastomotic disruption, or luminal obstruction. In the present case, several standard endoscopic strategies were considered but deemed unsuitable. The fistulous tract was located in the high cervical esophagus and was associated with a retroesophageal abscess cavity. Significant local inflammation and tissue friability reduced the likelihood of successful endoscopic clipping or suturing. Esophageal stenting was considered suboptimal due to the elevated risk of migration in the proximal cervical position and the limited capacity to control the associated extraluminal septic collection. Furthermore, classical intraluminal EndoVAC therapy would not have ensured adequate drainage of the retroesophageal abscess cavity. These anatomical and pathological factors justified the selection of an externally adapted extraluminal vacuum configuration.

Reported success rates of 81–88% refer predominantly to intrathoracic anastomotic leaks and postoperative esophageal perforations treated with intraluminal EndoVAC systems. Evidence specifically addressing cervical esophageal fistulas after Zenker surgery remains limited, and therefore direct extrapolation of these outcomes should be interpreted with caution [17].

Postoperative management of esophageal fistulas traditionally emphasizes aspiration prevention, infection control, and nutritional support [18,19]. While most EndoVAC protocols require strict nil per os status with enteral or parenteral nutritional supplementation [20], the extraluminal VAC technique employed in this case allowed controlled oral intake during treatment without compromising fistula healing. This represents a meaningful departure from established practice and underscores the functional benefits of preserving luminal integrity [21,22]. 

Hypopharyngeal fistulas following diverticulectomy represent a challenging postoperative complication [22]. Management strategies range from conservative treatment with antibiotics and nutritional support to surgical revision, endoscopic clipping, stenting, or vacuum therapy. The choice depends on fistula size, presence of abscess, tissue viability, and patient stability. In cases associated with extraluminal septic cavities, simple mucosal sealing may be insufficient, and active drainage becomes essential. 

Importantly, early and structured nutritional support has been consistently associated with improved fistula closure rates and survival outcomes [23,24]. By facilitating oral intake and reducing dependency on invasive feeding strategies, the external VAC approach may contribute to faster functional recovery and improved quality of life. Multidisciplinary collaboration among surgeons, endoscopists, nutrition specialists, and intensivists remains essential to maximize the therapeutic potential of this technique. In summary, as can be seen in Table 3, externally adapted VAC therapy represents an effective modification of classical EndoVAC treatment, offering a safe, flexible, and tissue-sparing alternative for the management of complex cervical esophageal fistulas.

This comparison is descriptive and hypothesis-generating. It does not imply superiority and is based on a single clinical observation.

The key indication for this modified approach appears to be the presence of a cervical esophageal fistula associated with an extraluminal abscess cavity that cannot be adequately managed by intraluminal drainage alone. Careful patient selection is essential, particularly in hemodynamically stable patients without generalized mediastinitis requiring immediate surgical re-exploration.

This report has several limitations. First, it describes a single-patient experience, which limits generalizability. Second, long-term follow-up data are not available to assess late recurrence or stricture formation. Third, no direct comparison with standard intraluminal EndoVAC therapy or alternative endoscopic approaches can be made. Therefore, conclusions regarding superiority or safety should be interpreted cautiously. Larger case series or comparative studies are required to better define the role of this adapted technique.

## 4. Conclusions

In conclusion, this case suggests that an externally adapted extraluminal vacuum-assisted closure configuration may represent a viable salvage option in carefully selected patients with complex cervical esophageal fistulas when conventional endoluminal strategies are not feasible. While the approach is conceptually consistent with established negative-pressure therapy principles, further experience and comparative data are required to better define its indications, safety profile, and long-term outcomes.

Overall, the case underscores the importance of individualized, multidisciplinary management in complex cervical esophageal fistulas and supports the potential role of externally applied VAC therapy as an innovative, minimally invasive alternative to traditional surgical or endoluminal interventions.

## Figures and Tables

**Table 1:** Laboratory parameters on first admission.

Laboratory Investigations	Values	Normal Values
Leukocytes	15.71 × 10^3^/µL	4.0–10.0 × 10^3^/µL
C-reactive protein	29.69 mg/dL	0–0.5 mg/dL
Neutrophils	81.3%	45.0–80.0%
Lymphocytes	8.2%	20.2–45.0%
Erythrocytes	3.62 × 10^6^/µL	3.8–5.8 × 10^6^/µL
Hemoglobin	10.9 g/dL	13.0–17.3 g/dL
Hematocrit	32.9%	39.0–51.0%

**Table 2:** Laboratory findings on fourth admission.

Laboratory Investigations	Values	Normal Values
Leukocytes	11.49 × 10^9^/L	4–10 × 10^9^/L
Neutrophils	9.13 × 10^9^/L	2–7 × 10^9^/L
Erythrocytes	3.71 × 10^12^/L	4.7–6.0 × 10^12^/L
Hemoglobin	11.1 g/dL	13.5–18.0 g/dL
Hematocrit	33.9%	37–54%
Platelets	317 × 10^9^/L	100–300 × 10^9^/L
C-reactive protein	224.7 mg/L	0–5.0 mg/L
Erythrocyte sedimentation rate (ESR)	120 mm/h	10–15 mm/h
Activated partial thromboplastin time (aPTT)	17.7 s	23–34 s
Prothrombin time (PT)	20.3 s	13.2–19.8 s
Fibrinogen	828 mg/dL	200–400 mg/dL
Urea	58 mg/dL	10–50 mg/dL

**Table 3:** Conceptual comparison between intraluminal EndoVAC and extraluminal cervical vacuum application in selected clinical scenarios.

Parameter	Classical EndoVAC Therapy	Externally Adapted VAC Therapy (Present Case)
Sponge placement	Intraluminal or transluminal, inside the esophageal lumen or fistula cavity	Extraluminal, positioned within the fistulous tract via a cervical approac
Access route	Endoscopic (transoral)	Surgical cervical access (laterocervical incision)
Endoscopic dependency	Requires repeated endoscopic placement and exchanges	Endoscopy used mainly for guidance; no repeated intraluminal manipulation
Effect on esophageal lumen	Partial or complete luminal occupation	Esophageal lumen preserved
Risk of mucosal/anastomotic injury	Present, especially with repeated sponge exchanges	Reduced, as no direct intraluminal contact
Suitability for cervical fistulas	Limited, technically challenging	Highly suitable
Management of associated abscess	Indirect drainage	Direct and controlled drainage of cervical/retropharyngeal abscess
Oral intake during therapy	Usually contraindicated (nil per os)	Liquid oral intake possible in selected cases
Need for alternative nutrition	Frequent (nasojejunal tube, jejunostomy, TPN)	Reduced dependency on invasive nutritional support
Indications	Esophageal leaks and fistulas accessible endoluminally	Complex cervical fistulas, unfavorable anatomy, failure or contraindication of endoscopic techniques
Patient comfort	Moderate; repeated endoscopies required	Improved; less invasive to the esophageal lumen
Clinical role	Established standard minimally invasive technique	Novel salvage and complementary approach
Main advantage	High closure rates, minimally invasive	Preserves lumen, allows oral intake, effective infection control
Main limitation	Not feasible in all anatomical settings	Requires surgical access and multidisciplinary expertise

VAC = Vacuum-Assisted Closure; TPN = Total Parenteral Nutrition.

## Data Availability

All original data from this study are included in the article; further information can be requested from the corresponding authors.
